# Supplementary material for: Dorsomedial prefrontal cortex links abstract planning to motor execution
Source: Commun Biol. 2026 Jun 23;9:1327. doi: 10.1038/s42003-026-10513-4 (PMC13655160; doi:10.1038/s42003-026-10513-4)
Supplement: Supplementary file 1 [file 42003_2026_10513_MOESM1_ESM.pdf]

Dorsomedial prefrontal cortex links abstract planning to motor execution

Ricardo J. Alejandro, Iris Ikink, Emmanouela Foinikianaki, Clay B. Holroyd

Department of Experimental Psychology, Ghent University, Ghent, Belgium

SUPPLEMENTARY INFORMATION

Table S1. Stimuli for task “catch” trials

Stimuli	Code<br>shape | color | shape | color
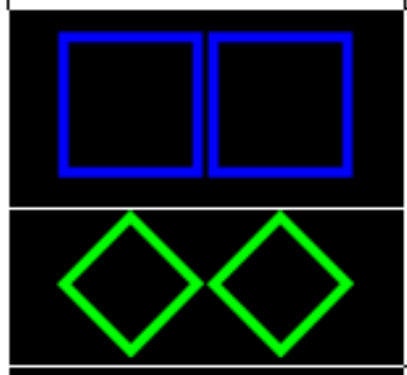	#####
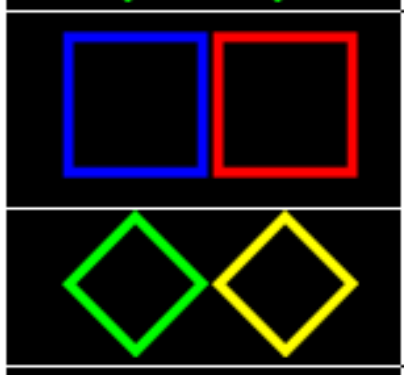	####&
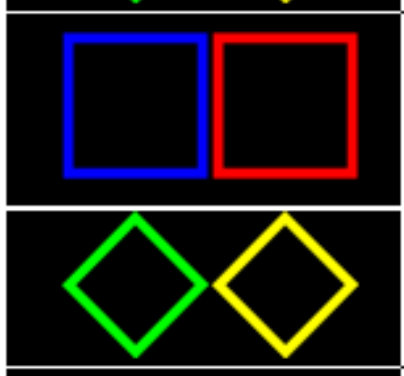	####&
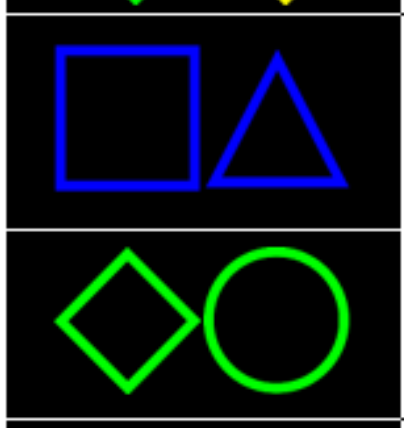	##&#
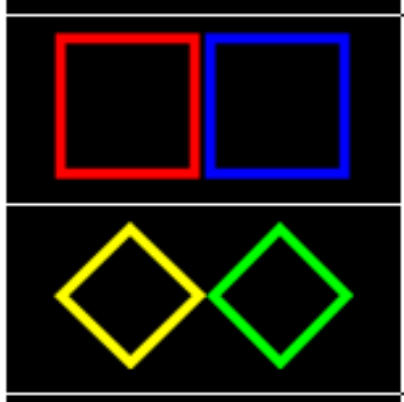	#&##
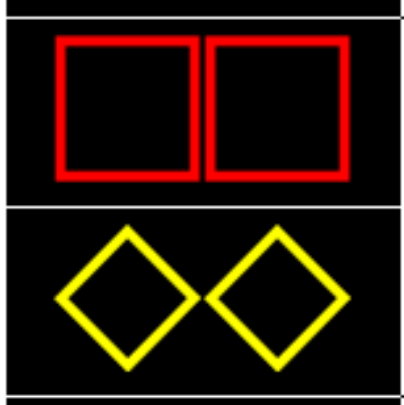	#&#&
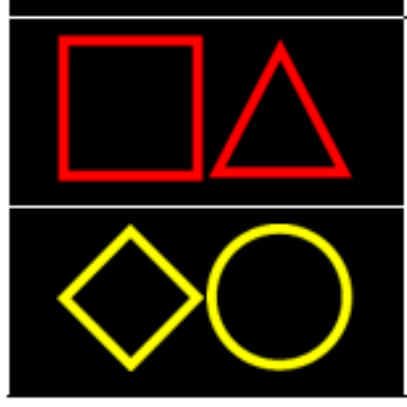	#&&&

Stimuli	Code<br>shape | color | shape | color
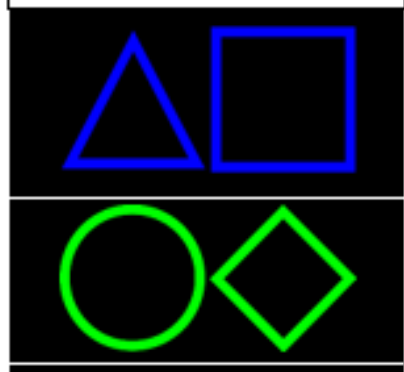	&####
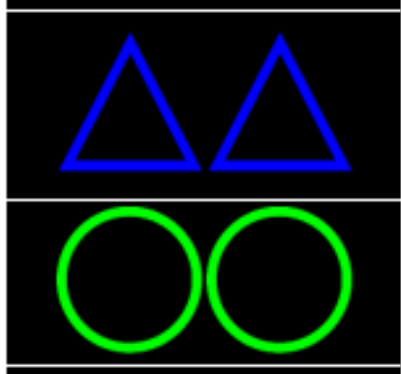	&#&#
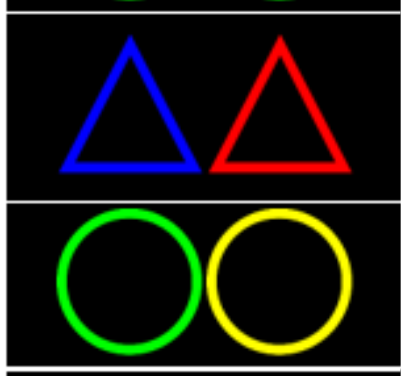	&#&&
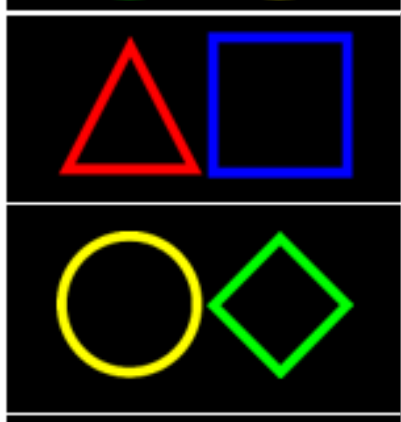	&&##
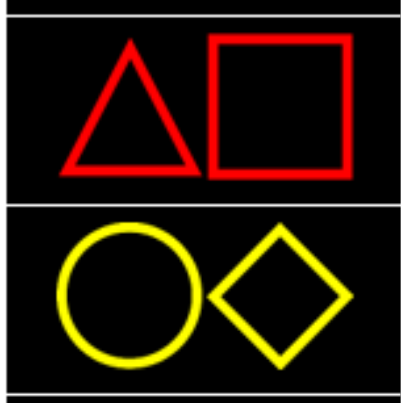	&&#&
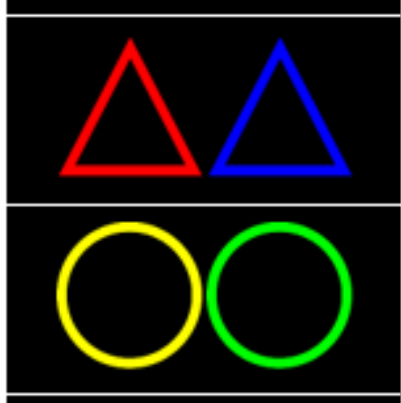	&&&#
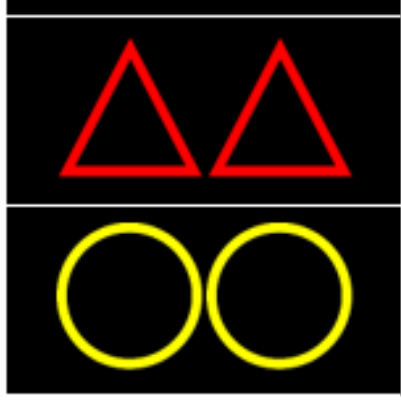	&&&&

Table S2. Reaction time analysis – main fMRI task

Results of the Reaction Time (RT) analysis of the behavioral data during the fMRI task. Factors were sequence step (1, 2, 3, 4), planning duration (short, medium, long), and their interaction.

	Sum Sq	Mean Sq	NumDF	DenDF	F value	Pr(>F)
step	1418.81	472.94	3	32907	24256.561	**< 2.2e-16**
plan duration	1.47	0.73	2	32908	37.644	**< 2.2e-16**
step:plan duration	6.51	1.09	6	32907	55.691	**< 2.2e-16**

Table S3. Post-hoc pairwise differences – main fMRI task

Subsequent post-hoc (Tukey HSD) tests performed to identify the direction of significant effects and interactions from the RT analysis of the behavioral data during the fMRI task.

contrast	estimate	SE	df	t.ratio	p.value
**Main effect: sequence step**					
step1 - step2	0.45770	0.00218	32907	210.402	**<.0001**
step1 - step3	0.46169	0.00218	32907	212.236	**<.0001**
step1 - step4	0.51072	0.00218	32907	234.778	**<.0001**
step2 - step3	0.00399	0.00218	32907	1.833	0.2576
step2 - step4	0.05302	0.00218	32907	24.375	**<.0001**
step3 - step4	0.04904	0.00218	32907	22.542	**<.0001**
**Main effect: plan duration**					
long - med	-0.00685	0.00189	32907	-3.625	**0.0008**
long - short	-0.01629	0.00189	32908	-8.633	**<.0001**
med - short	-0.00944	0.00189	32908	-5.034	**<.0001**
**Interaction: sequence step X plan duration**					
step = 1:					
long - med	-0.031884	0.00378	32907	-8.434	**<.0001**
long - short	-0.075597	0.00377	32907	-20.032	**<.0001**
med - short	-0.043713	0.00375	32907	-11.658	**<.0001**
step = 2:					
long - med	0.003342	0.00378	32907	0.884	0.6504
long - short	0.007083	0.00377	32907	1.877	0.1454
med - short	0.003741	0.00375	32907	0.998	0.5783
step = 3:					
long - med	0.001336	0.00378	32907	0.353	0.9334
long - short	0.002837	0.00377	32907	0.752	0.7325
med - short	0.001501	0.00375	32907	0.400	0.9155
step = 4:					
long - med	-0.000210	0.00378	32907	-0.056	0.9983
long - short	0.000508	0.00377	32907	0.135	0.9901
med - short	0.000718	0.00375	32907	0.191	0.9800

Figure S1. Reaction time (RT) results – behavioral training.

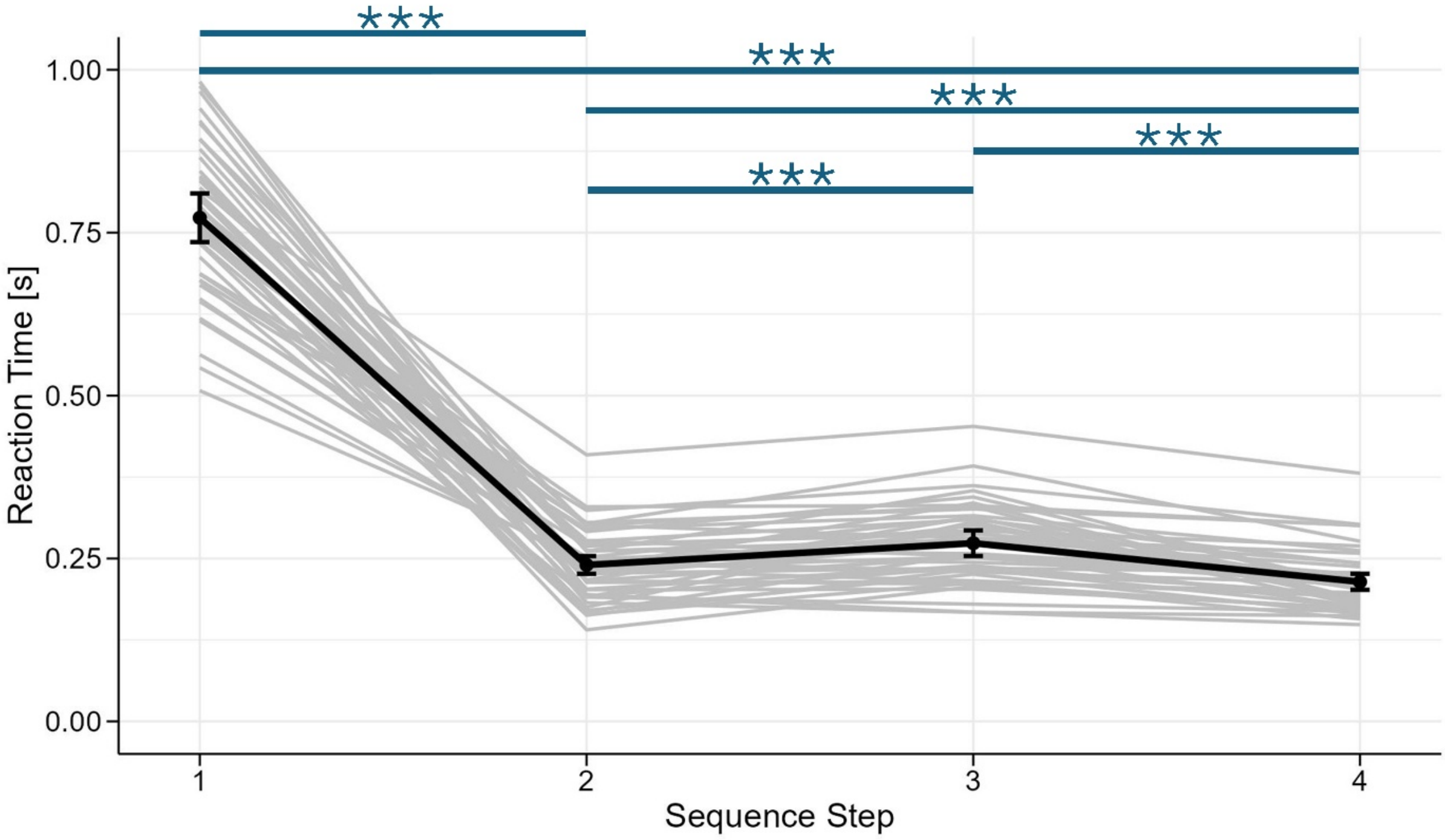

Horizontal lines represent post-hoc pairwise comparisons following the main effect of sequence step on RT. Bold black line represents the average RT across participants, gray lines represent individual participants. Asterisks denote statistical significance at $p < 0.0001$.

Figure S2. Post-hoc pairwise difference for the main effect of plan duration – main fMRI task.

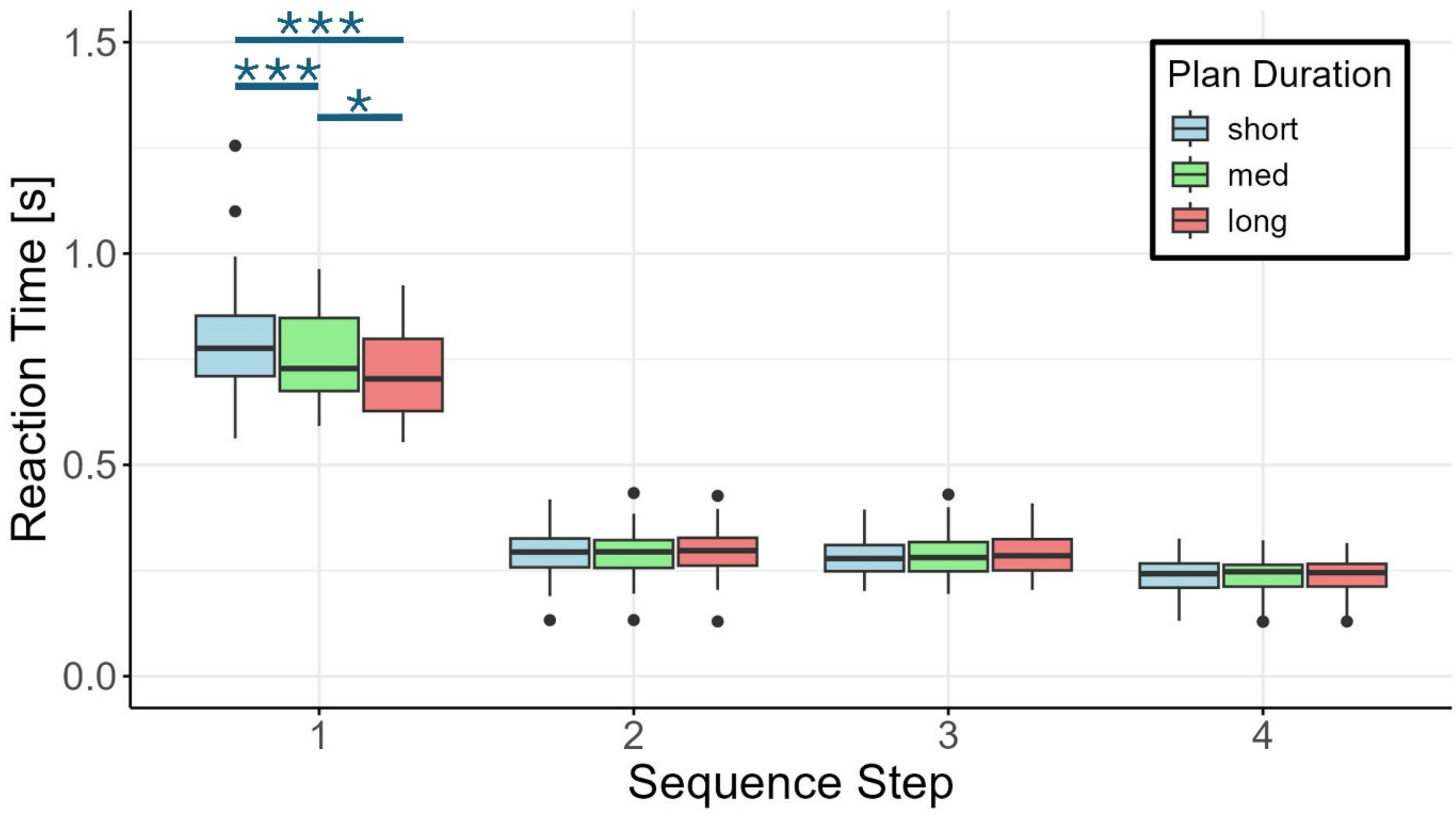

Figure S3. Collinearity assessment: correlation between regressors of interest and VIF

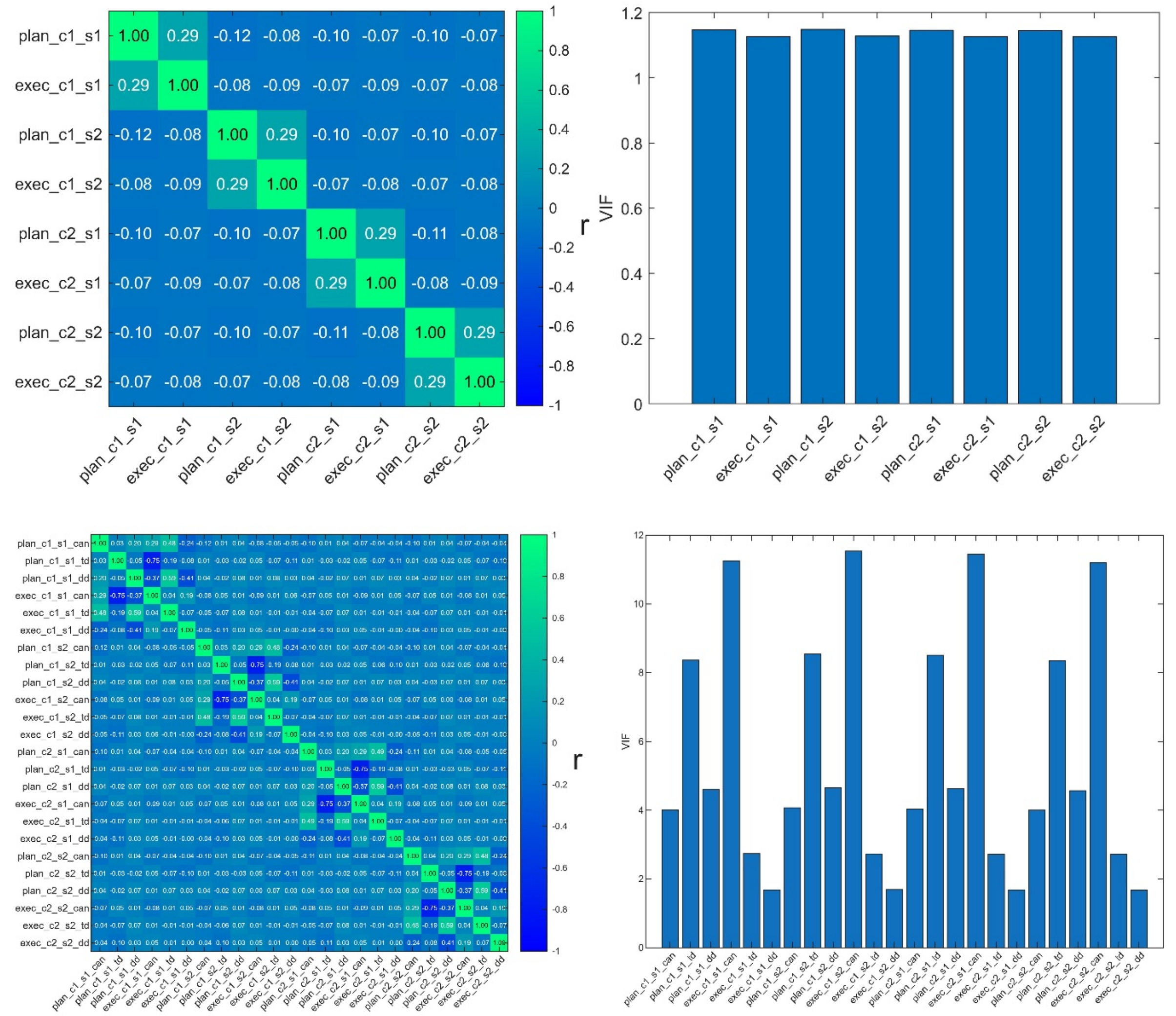

Our primary analysis relied on training a classifier on activations from the planning phase and testing it on activity from the execution phase. To evaluate that these regressors were not problematically correlated and sufficiently independent for a successful cross-decoding analysis, we evaluated the collinearity in our regressors of interest. For each subject, we extracted the filtered and whitened design matrix from their GLM specification (see main text methods). Within each experimental run, we computed two metrics for the eight regressors of interest (four planning and four execution regressors for conditions c1s1, c1s2, c2s1, c2s2, respectively): a full regressor (8x8) correlation matrix (left panel), from which we could assess the relationship of each regressor to the rest, and the variance inflation factor (VIF [1]) for each regressor (right panel). The VIF of regressor X_i is defined as $VIF_i = 1/(1 - R_i^2)$, where R_i^2 is the coefficient of determination obtained by regressing X_i on all other regressors in the model ($X_i = \beta_1 X_1 + \cdots + \beta_{i-1} X_{i-1} + \beta_{i+1} X_{i+1} + \cdots + \beta_N X_N + \epsilon$) [1,2]. The VIF provides a direct measure of collinearity since its value indicates the degree at which one regressor represents a linear combination of the other regressors [2], a property that pairwise correlations might not always capture. Higher values of VIF denote higher presence of collinearity, with values greater than 5 or 10 considered problematic [2] (but see [3]). These two metrics were calculated at the run level before being averaged within each subject to create stable per-subject and group-level estimates. As expected, the VIF was low for all regressors of interest (mean = 1.14, SD = 0.03; top panel, right), the correlations were moderate between planning and execution regressors from the same condition (mean = 0.29, SD = 0.04; top panel, left) and low between planning and execution regressors from different conditions (mean = -0.07, SD = 0.017; top panel, left).

We repeated the same procedure on the full 24-regressor-per-run design, i.e. the eight conditions described above each modelled with three basis functions (canonical HRF, time derivative, dispersion derivative). For each run we computed the 24 x 24 regressor-correlation matrix and the corresponding VIF for each regressor, averaged these across runs and then across subjects. The mean VIF (across all 24 regressors; bottom panel, right) was 5.47 (SD = 3.46). For the canonical regressors the mean VIF was 7.69 (SD = 3.82). For the time-derivative regressors the VIF was 5.58 (SD = 2.96), and 3.15 (SD = 1.49) for the dispersion-derivative regressors. The inflation of the canonical VIFs in this extended design is not task-driven, for instance the temporal derivative, once convolved with the event sequence, becomes correlated with a slightly shifted canonical regressor for the same condition. This is a known property of SPM's informed basis set and does not reflect collinearity between task conditions, which remains low as reported in the canonical regressors only analysis (see above).

Table S4. Activation value maps for the Multivariate Pattern Analyses.

Activation values for key brain regions (clusters) identified during multivariate analyses: searchlight cross-decoding from planning to execution, and searchlight RSA comparing all 4 conditions during the planning phase.

Region	voxels	Peak t	MNI coords (peak)			Cluster p
			x	y	z	
**Searchlight-cross-decoding planning → execution (abstract)**						
Lenticular nucleus, Pallidum, left<br>Lenticular nucleus, Putamen, left	29	5.49	-26	-14	-4	0.071 (uncorr.)
Superior frontal gyrus, medial, right<br>Medial frontal gyrus, right	26	4.22	10	46	40	0.098 (uncorr.)
Middle frontal gyrus, left	24	3.94	-48	30	30	0.098 (uncorr.)
Inferior frontal gyrus, triangular part, left		3.60	-42	36	24	
**Searchlight-cross-decoding planning → execution (perceptual)**						
Calcarine fissure and surrounding cortex, right	107	4.80	4	-76	10	0.046 (uncorr.)
Calcarine fissure and surrounding cortex, left		4.04	-10	-76	10	
**Searchlight-RSA, planning (abstract)**						
Superior frontal gyrus, medial, right<br>Medial frontal gyrus, right	46	4.27	3	38	44	0.014 (FWE)
		3.91	6	47	44	
Superior frontal gyrus, medial, left<br>Medial frontal gyrus, left		4.23	-3	50	44	
**Searchlight-RSA, planning (perceptual)**						
Middle occipital gyrus, left<br>Superior occipital gyrus, left	120	6.12	-18	-91	1	0.000 (FWE)

Inferior occipital gyrus, left<br>Calcarine fissure and surrounding cortex, left<br>Lingual gyrus, left<br>Fusiform gyrus, left		4.66	-12	-97	11	

Figure S4. ROI analyses.

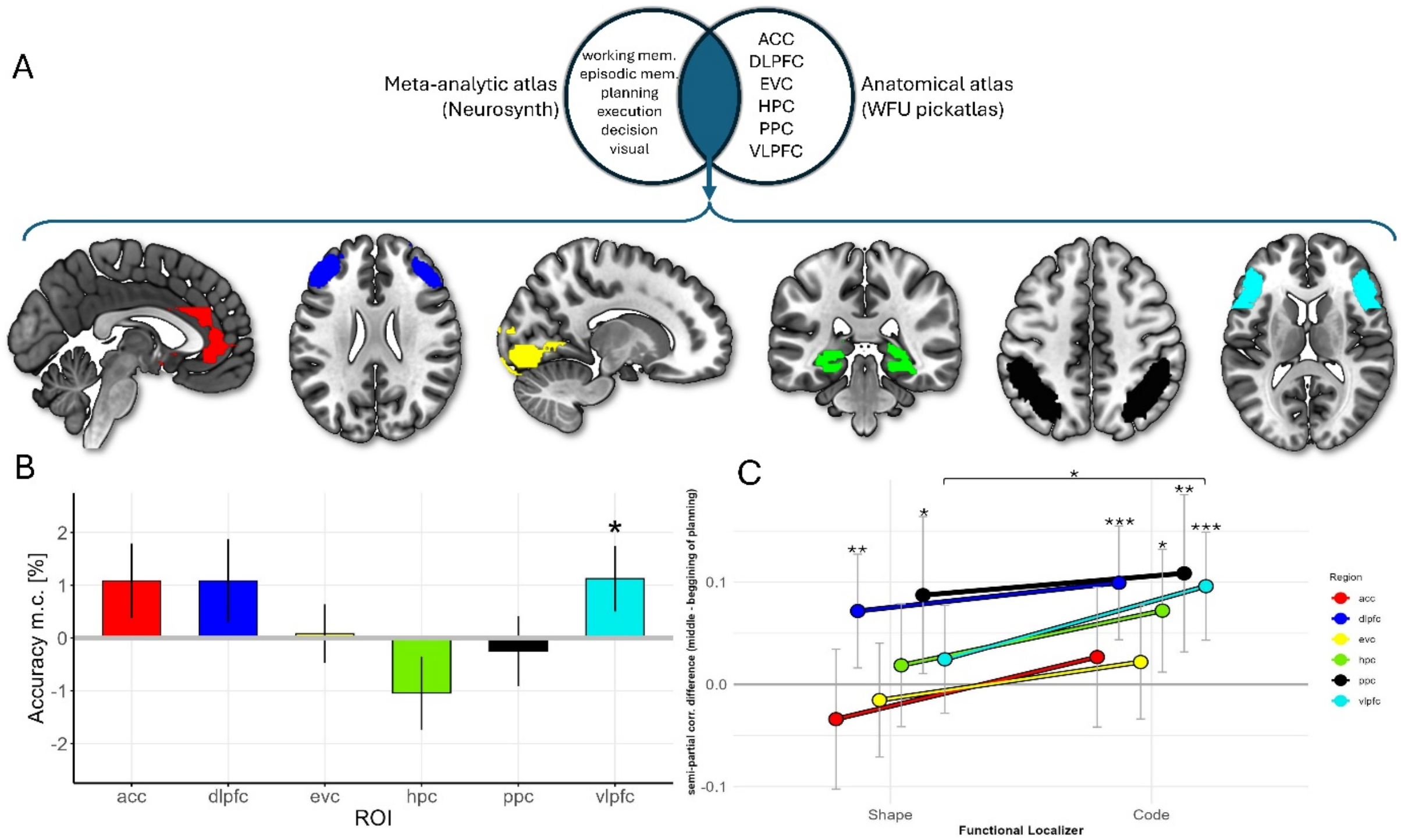

A. ROI selection. As pre-registered, we ran follow-up analyses in specific regions of interest (ROI) for two reasons: 1) our main hypothesis specified that a higher-order context will be represented in planning-related regions, and 2) previous literature has shown that decoding analyses tend to be relatively stringent, especially with regards to information in prefrontal cortex [4]. We selected planning-related ROIs located in: (rostral) anterior cingulate cortex (ACC), ventrolateral prefrontal cortex (VLPFC), dorsolateral prefrontal cortex (DLPFC), posterior parietal cortex (PPC), hippocampus (HPC), and early visual cortex (EVC) as control region. First, a binary mask of each anatomical region was defined, extracted, and combined using the WFU pickatlas software [5] (version 3.0.5). Second, from the metanalytical tool Neurosynth (https://neurosynth.org/analyses/), we extracted the resulting (FDR-corrected) masks from the search terms “working memory”, “episodic memory”, “planning”, “execution”, “decision”, and “visual.” These metaanalytically-defined masks were binarized and combined into one single aggregate mask. Finally, each anatomically-defined ROI mask was intersected with the aggregate mask, resulting in 6 ROIs. Each ROI mask was registered back to each subject’s native space via estimation of inverse deformation fields. **B. ROI-decoding results.** Decoding was significantly above chance for VLPFC. **C. Semi-partial correlation results.** Abstract representations increased significantly from the beginning to the middle of planning in DLPFC, HPC, PPC, and VLPFC. Perceptual representations increased significantly from the beginning to the middle of planning in DLPFC and PPC. The dynamics between abstract and perceptual representations were significantly different only for VLPFC. ROI-RSA results were not significant in any of the ROIs.

SUPPLEMENTARY REFERENCES

1. Mumford, J. A. *et al.* Unintended bias in the pursuit of collinearity solutions in fMRI analysis. *Imaging Neurosci.* **3**, IMAG.a.958 (2025).
2. Mumford, J. A., Poline, J.-B. & Poldrack, R. A. Orthogonalization of Regressors in fMRI Models. *PLOS ONE* **10**, e0126255 (2015).
3. O'brien, R. M. A Caution Regarding Rules of Thumb for Variance Inflation Factors. *Qual. Quant.* **41**, 673–690 (2007).
4. Bhandari, A., Gagne, C. & Badre, D. Just above Chance: Is It Harder to Decode Information from Prefrontal Cortex Hemodynamic Activity Patterns? *J. Cogn. Neurosci.* **30**, 1473–1498 (2018).
5. Maldjian, J. A., Laurienti, P. J., Kraft, R. A. & Burdette, J. H. An automated method for neuroanatomic and cytoarchitectonic atlas-based interrogation of fMRI data sets. *NeuroImage* **19**, 1233–1239 (2003).
