# Supplementary material for: Dorsomedial prefrontal cortex links abstract planning to motor execution
Source: Commun Biol. 2026 Jun 23;9:1327. doi: 10.1038/s42003-026-10513-4 (PMC13655160; doi:10.1038/s42003-026-10513-4)
Supplement: Supplementary file 2 [file 42003_2026_10513_MOESM2_ESM.pdf]

nature portfolio

Corresponding author(s): Ricardo Alejandro

Last updated by author(s): May 20, 2026

Reporting Summary

Nature Portfolio wishes to improve the reproducibility of the work that we publish. This form provides structure for consistency and transparency in reporting. For further information on Nature Portfolio policies, see our Editorial Policies and the Editorial Policy Checklist.

Statistics

For all statistical analyses, confirm that the following items are present in the figure legend, table legend, main text, or Methods section.

n/a	Confirmed	
☐	☒	The exact sample size (*n*) for each experimental group/condition, given as a discrete number and unit of measurement
☐	☒	A statement on whether measurements were taken from distinct samples or whether the same sample was measured repeatedly
☐	☒	The statistical test(s) used AND whether they are one- or two-sided<br>*Only common tests should be described solely by name; describe more complex techniques in the Methods section.*
☐	☒	A description of all covariates tested
☐	☒	A description of any assumptions or corrections, such as tests of normality and adjustment for multiple comparisons
☐	☒	A full description of the statistical parameters including central tendency (e.g. means) or other basic estimates (e.g. regression coefficient) AND variation (e.g. standard deviation) or associated estimates of uncertainty (e.g. confidence intervals)
☐	☒	For null hypothesis testing, the test statistic (e.g. *F*, *t*, *r*) with confidence intervals, effect sizes, degrees of freedom and *P* value noted<br>*Give P values as exact values whenever suitable.*
☒	☐	For Bayesian analysis, information on the choice of priors and Markov chain Monte Carlo settings
☒	☐	For hierarchical and complex designs, identification of the appropriate level for tests and full reporting of outcomes
☐	☒	Estimates of effect sizes (e.g. Cohen's *d*, Pearson's *r*), indicating how they were calculated

Our web collection on statistics for biologists contains articles on many of the points above.

Software and code

Policy information about availability of computer code

Data collection	Psychopy 2022.2.4<br>Pavlovia<br>Siemens syngo MR platform
Data analysis	SPM12, The Decoding Toolbox version 3.99F, rsatoollbox, all running under Matlab R2023a<br>R 4.4.0 running under RStudio 2024.09.1+394

For manuscripts utilizing custom algorithms or software that are central to the research but not yet described in published literature, software must be made available to editors and reviewers. We strongly encourage code deposition in a community repository (e.g. GitHub). See the Nature Portfolio guidelines for submitting code & software for further information.

Data

Policy information about availability of data

All manuscripts must include a data availability statement. This statement should provide the following information, where applicable:

- Accession codes, unique identifiers, or web links for publicly available datasets
- A description of any restrictions on data availability
- For clinical datasets or third party data, please ensure that the statement adheres to our policy

Data and code will be made available upon acceptance for publication.

Research involving human participants, their data, or biological material

Policy information about studies with human participants or human data. See also policy information about sex, gender (identity/presentation), and sexual orientation and race, ethnicity and racism.

Reporting on sex and gender	Sex and gender were not considered in study design. In the manuscript we only referred to the sex by using the words 'female' and 'male'
Reporting on race, ethnicity, or other socially relevant groupings	We did not report on race, ethnicity, or other socially relevant groupings
Population characteristics	We reported on sex and age
Recruitment	Participants were recruited through the SONA research participation system within the Ghent University network and through social media
Ethics oversight	The Ethics Committee of Ghent University Hospital reviewed and approved this study

Note that full information on the approval of the study protocol must also be provided in the manuscript.

Field-specific reporting

Please select the one below that is the best fit for your research. If you are not sure, read the appropriate sections before making your selection.

☐ Life sciences ☒ Behavioural & social sciences ☐ Ecological, evolutionary & environmental sciences

For a reference copy of the document with all sections, see nature.com/documents/nr-reporting-summary-flat.pdf

Behavioural & social sciences study design

All studies must disclose on these points even when the disclosure is negative.

Study description	Quantitative experimental human neuroimaging study. Participants performed a controlled behavioral task during functional MRI scanning, and quantitative behavioral (reaction time and accuracy) and neuroimaging data (multivariate fMRI activation patterns) were analyzed using statistical modeling, multivariate pattern analysis, and representational similarity analysis.
Research sample	Ghent University undergraduates, 35 females, mean age = 24.4, range = 18.8 – 37.5
Sampling strategy	Participants were recruited using a convenience sampling strategy from the local university community via the SONA participant recruitment system. Individuals were screened for MRI compatibility and normal or corrected-to-normal vision prior to participation. All eligible participants who successfully completed the required online training session were invited to participate in the subsequent fMRI experiment.
Data collection	We conducted a two-part experiment. In the first part, participants underwent behavioral training online, which was intended for participants to learn the task stimuli and rules.<br>For the second part, participants underwent fMRI scanning while performing a slightly modified version of the task plus two additional functional localizer tasks inside an fMRI scanner. The two portions of the experiment were completed by the same set of participants.
Timing	Data were collected between May and July of 2024
Data exclusions	The data of two participants were removed due to failure to complete the training (online training timed out), and the data of one other participant were removed due to MRI-incompatibility
Non-participation	No participants dropped-out/declined participation
Randomization	Participants were not allocated into specific experimental groups

Reporting for specific materials, systems and methods

We require information from authors about some types of materials, experimental systems and methods used in many studies. Here, indicate whether each material, system or method listed is relevant to your study. If you are not sure if a list item applies to your research, read the appropriate section before selecting a response.

Materials & experimental systems

n/a	Involved in the study
☒	☐ Antibodies
☒	☐ Eukaryotic cell lines
☒	☐ Palaeontology and archaeology
☒	☐ Animals and other organisms
☒	☐ Clinical data
☒	☐ Dual use research of concern
☒	☐ Plants

Methods

n/a	Involved in the study
☒	☐ ChIP-seq
☒	☐ Flow cytometry
☐	☒ MRI-based neuroimaging

Plants

Seed stocks

Report on the source of all seed stocks or other plant material used. If applicable, state the seed stock centre and catalogue number. If plant specimens were collected from the field, describe the collection location, date and sampling procedures.

Novel plant genotypes

Describe the methods by which all novel plant genotypes were produced. This includes those generated by transgenic approaches, gene editing, chemical/radiation-based mutagenesis and hybridization. For transgenic lines, describe the transformation method, the number of independent lines analyzed and the generation upon which experiments were performed. For gene-edited lines, describe the editor used, the endogenous sequence targeted for editing, the targeting guide RNA sequence (if applicable) and how the editor was applied.

Authentication

Describe any authentication procedures for each seed stock used or novel genotype generated. Describe any experiments used to assess the effect of a mutation and, where applicable, how potential secondary effects (e.g. second site T-DNA insertions, mosiacism, off-target gene editing) were examined.

Magnetic resonance imaging

Experimental design

Design type

Task, event-related design

Design specifications

Each participant completed 4 main-task fMRI blocks (runs) of approximately 10 minutes each
Each main-task run contained 44 trials, with each trial lasting 13 seconds
Within each trial, phases were jittered: fixation and stimulus presentation were each 2.0 ± 0.5 s, the planning delay was 6.0 ± 1.5 s, and the execution phase was 3.0 ± 0.5 s; incorrect trials received 0.5 s feedback

After the main task, participants completed two functional localizer tasks (shape and code) lasting approximately 10 minutes each
The shape localizer task included 68 task trials, including 15 trials per standard-trial shape-pair (or 30 trials per each of the two standard-trial codes), and 8 (~12%) catch trials. All the trials in the shape localizer task lasted for 8.75 s
The code localizer task included 68 task trials (30 trials per each of the two standard-trial codes) and 8 (~12%) catch trials. All the trials in the shape localizer task lasted for 9.25 s

Behavioral performance measures

Behavioral performance was assessed using reaction times (RTs) and accuracy. RTs were measured for each of the four sequential responses within each trial (RT1–RT4) and analyzed as a function of sequence step and planning duration. Accuracy was computed as the proportion of correct trials (correct 4-character code entry), and accuracy was summarized across blocks.

Acquisition

Imaging type(s)

structural and functional

Field strength

3 T

Sequence & imaging parameters

Using a scanner Siemens MAGNETOM PrismaFit 3T MRI with a 64-channel head coil, for each of our participants we collected:
a high-resolution T1-weighted anatomical scan using an MPRAGE sequence (176 slices; voxel size = 1 mm isotropic; TR = 2250 ms; TE = 4.18 ms; FoV = 789 256 mm), and
whole-brain, T2*-weighted multi-band echo-planar imaging (EPI) functional scans (54 slices per volume; TR = 1780 ms; TE = 27 ms; voxel size = 2.5 mm isotropic; FoV = 210 mm; interleaved slice acquisition).

Area of acquisition

whole-brain

Diffusion MRI ☐ Used ☒ Not used

Preprocessing

Preprocessing software	fMRIPrep 23.2.3
Normalization	Individual analyses were performed on unsmoothed, unnormalized data<br><br>For group analyses, volume-based spatial normalization to one standard space was performed through nonlinear registration
Normalization template	ICBM 152 Nonlinear Asymmetrical template version 2009c (TemplateFlow ID: 828 MNI152NLin2009cAsym)
Noise and artifact removal	The T1w image was corrected for intensity non-uniformity (INU) with N4BiasFieldCorrection distributed with ANTs 2.5.0 and used as T1w-reference throughout the workflow
Volume censoring	*Define your software and/or method and criteria for volume censoring, and state the extent of such censoring.*

Statistical modeling & inference

Model type and settings	Subject-level analyses were conducted using a general linear model (GLM) to estimate beta values for each condition of interest (planning and execution phases, separated by stimulus/code conditions). Beta estimates from the first-level GLM were used as inputs for multivariate decoding and RSA analyses.<br><br>Searchlight MVPA used a support vector machine (SVM) classifier. A cross-decoding procedure trained the classifier on planning-phase activity patterns and tested on execution-phase activity patterns using leave-one-run-out cross-validation.<br><br>Searchlight RSA compared neural representational dissimilarity matrices (RDMs) derived from planning-phase beta patterns against hypothesis-driven abstract and perceptual model RDMs.
Effect(s) tested	The primary effects tested were neural representations of abstract task context during planning and their generalization to execution. Specifically, cross-decoding analyses tested whether multivariate activity patterns during the planning phase could predict activity patterns during the execution phase for the same abstract codes across different stimuli. Representational similarity analyses tested whether planning-phase activity patterns reflected abstract task relationships versus perceptual similarity.

Specify type of analysis: ☒ Whole brain ☐ ROI-based ☐ Both

Statistic type for inference<br><br>(See Eklund et al. 2016)	Group-level inference was based on cluster-wise statistical tests of decoding accuracy and representational similarity values derived from searchlight analyses.
Correction	Whole-brain imaging results were corrected for multiple comparisons using family-wise error (FWE) correction at the cluster level ($p < 0.05$). Exploratory analyses additionally reported results at an uncorrected threshold of $p < 0.001$.

Models & analysis

n/a	Involved in the study
☒	☐ Functional and/or effective connectivity
☒	☐ Graph analysis
☐	☒ Multivariate modeling or predictive analysis

Multivariate modeling and predictive analysis	Multivariate modeling used searchlight-based support vector machine (SVM) classifiers to perform cross-decoding analyses. Classifiers were trained on planning-phase activity patterns and tested on execution-phase patterns using leave-one-run-out cross-validation. Representational similarity analysis (RSA) used searchlight approaches to compare neural representational dissimilarity matrices with hypothesis-driven abstract and perceptual models.
